# Supplementary material for: Are carbon emissions trading and green financial instruments synergistic? -Comprehensive quantitative research based on content analysis
Source: PLoS One. 2024 Mar 7;19(3):e0298601. doi: 10.1371/journal.pone.0298601 (PMC10919601; doi:10.1371/journal.pone.0298601)
Supplement: S1 Data — (ZIP) [file pone.0298601.s001.zip › Data/地级市Z_F工作报告.zip!/╕ú╜¿/─╧╞╜╩╨/└┤╫╘╣1⁄2╓┌║┼í╛┬φ┐╦╩2╛▌═°í┐_2008.docx]

各位代表：

现在，我代表南平市人民政府向大会报告工作，请予审议，并请市政协委员和其他列席人员提出意见。

一、海西绿色腹地建设富有成效

2007年，闽北风调雨顺，跃升发展。在省委、省政府和市委正确领导下，在市人大和市政协监督支持下，我市各级政府认真贯彻党的十七大精神，坚持科学发展，抢抓海西历史机遇，加快绿色腹地建设，市三届人大一次会议确定的年度目标全面实现。预计全年实现生产总值474亿元，增长15%%，是十年来增幅最高的年份。规模以上工业产值417亿元，增长26.5%%；农林牧渔产值192.5亿元，增长6.5%%。三次产业结构为25.3:37.3:37.4，二产比重提高1个百分点。财政总收入40.63亿元，增长29.81%%，提前三年完成“十一五”目标；税性比重达82.47%%，提高2.55个百分点；财政收入占GDP的8.57%%，提高0.61个百分点。全社会固定资产投资298亿元，增长38%%。实施重点项目94个，完成投资80.2亿元，增长36.2%%。社会消费品零售总额174.4亿元，增长15.6%%。银行存款增长3.6%%，贷款增长23.1%%。全市跃升发展态势显现，本届政府届首之年开局良好。

绿色腹地内涵深化。主动融入海西建设大局，认真审视闽北的优势、作用和前景，进一步丰富绿色腹地的内涵，进一步发挥南接北联战略通道的作用，进一步突显纵深推进前锋平台的地位。在融入海西的大局中，发展观念逐步转变，没地、没钱、信心不足的心态逐步扭转，不等、不靠、不让的工作状态逐步形成。着眼当前和长远发展，展开城建、金融、工业、旅游和新农村建设等系列调研，把海西绿色腹地建设战略具体化，逐项跟进配套政策，逐项推动落实，经济发展速度加快、质量提升、后劲增强。

战略重点全面展开。按照“一点一线一重心”战略布局，加快工业园区、县域经济和中心城市建设。闽北产业集中区童游机械电子组团和林产工贸园区完成基础设施投资2.17亿元，入园企业44家，总投资8.42亿元；整合组建南平工业园区，完成基础设施投资2.68亿元，新入园企业16家，总投资8.72亿元；荣华山产业组团完成总体规划并开工建设。邵武经济开发区、建瓯笋竹城等县市园区形成规模，各具特色。南平中心城市建设力度加大，首批26个城建重点项目开始实施。沿江快速通道和景观带项目建设全面展开，黄墩大桥、剑州大桥、西城大桥加快建设，江南公园一、二期工程顺利竣工，八仙小区开发初具规模，市区第二水源工程全面开工，昼锦、延福排涝站投入运行。

产业支撑不断增强。修订工业发展扶持政策，出台扶持重点工业企业发展的若干意见，确定培育首批31家新“五南”式企业。七大产业实现产值246亿元，增长30.5%。一批重点企业实施重大技改和扩建项目，工业项目投资占重点项目投资比重35.9%，同比提高16.7个百分点。全市产值上亿元企业达65家，新创国家级品牌4个、省级品牌32个，工业支撑力和带动力明显增强。旅游产业发展加快，接待人数、旅游总收入分别增长16.2%和21.5%，是“十五”以来增速最快的年份。对外经贸不断拓展，“5·13”投资洽谈会、朱子文化节和“6·18”项目成果对接会、“9·8”贸洽会等活动成效明显，全年实际利用外资4.18亿美元，增长10%%；外贸出口4.95亿美元，增长15.7%；山海协作项目实际到资99.16亿元，增长62.3%。

农村工作扎实有效。落实支农惠农政策，实施“十个到村”，新农村建设全面展开。农业产业化加快推进，全市规模以上农业产业化企业554家，增加60家；农村专业合作经济组织814个，增加85个。深化农村集体林权制度改革，实施生态公益林管护、林权抵押贷款等配套措施，林权登记发证面积1853.6万亩，占应发证面积的94.8%。农村公路硬化1306公里，新建农村客运站20个、候车亭360个，行政村通客车率93%。建设林业“四大基地”40.8万亩。新建竹山机耕路2451.6公里，改造中低产田9.36万亩，投入烟叶基础设施1.42亿元。推进农村信息化建设，行政村宽带覆盖率达70%。开展“家园清洁行动”，新建户用沼气池8000口。农村工作机制进一步完善，新下派506名村支书，选派科技特派员647名、流通助理77名，选聘农村“八大员”15897人。164个部门与118个村结成互动联动关系。

基础设施加快建设。浦南高速公路年底可建成通车，武邵高速公路全线开工，宁武高速公路工程可行性研究进入审批阶段，松建高速公路工程可行性研究上报国家审查，龙浦、建古、邵光、南平绕城等高速公路前期工作有序推进。向莆铁路南平连接线近期可开工，峰福线横南段电气化技改项目年内将开工建设，南平城区铁路总体规划基本完成，南平东支线改线完成工程可行性研究。南平500千伏输变电工程开工，220千伏、110千伏输变电工程7项竣工投产、10项正在施工。顺昌洋口电站3台机组并网发电。核电项目完成初步可行性研究。

社会事业稳步发展。全面落实农村义务教育阶段学生免除学杂费、免交课本费和寄宿制学生生活补助政策，实施中职学校助学金制度。改造农村中小学危房7.78万平方米。武夷学院正式挂牌并招收首批本科生，武夷山职业学院正式招生，江南职教园区启动建设。实施国家、省级科技项目39项，稳步推进省级可持续发展实验区建设，科技服务向生产第一线延伸。着力打造文化品牌，公共文化服务持续推进，南平大剧院、博物馆、南武体育馆等项目建设进入扫尾阶段。广播、电视综合覆盖率分别达95.8%、97.8%。群众体育运动蓬勃开展，竞技体育取得新成绩。公共医疗卫生体系进一步完善，4个县达到农村初级卫生保健合格县标准，建成社区卫生服务机构36个，改造乡镇卫生院26所，实现村村有卫生所。落实计生奖励扶助政策，持续稳定低生育水平，人口自然增长率6.57‰。人事编制、民族宗教、防灾减灾、档案方志、妇女儿童、老区、老龄、残疾人等事业有新发展。

民生水平持续提高。城镇居民人均可支配收入13161.5元，增长16.6%%；农民人均纯收入5019元，增长13.5%%。新增城镇就业2.4万人、下岗失业人员再就业1.4万人、农村劳动力转移就业5万人，城镇登记失业率3.68%%。城乡低保实现应保尽保，农村低保年人均标准从1000元提高到1200元，城镇低保月人均补差从58元提高到87元，保障人数达11.39万人。全面实施新型农村合作医疗，参合农民203.83万人。城镇居民基本医疗保险列入全国试点，已参保16.34万人。南平中心城区开发建设廉租住房9000平方米146套，发放廉租住房补贴10万元。新建村级供水工程226个，改善14.53万人饮水条件。实施“造福工程”搬迁4900人。新建和改建15所农村敬老院。全面落实大中型水库移民后期扶持政策。筹建南平慈善总会。市委、市政府确定的18件为民办实事项目全面完成。

和谐社会加快构建。自觉接受人大的法律监督、工作监督和政协的民主监督，办理市人大代表议案和建议207件、市政协委员提案222件，办复率均为100%%。密切与民主党派、工商联、无党派人士和人民团体的联系，主动听取各界人士的意见和建议。依法治市和依法行政积极推进，机关效能建设深入开展，政府绩效评估进一步强化。加强反腐倡廉工作，落实廉洁自律各项规定，坚持政府部门述职述廉制度，纠风工作不断加强，治理商业贿赂取得新成效。深入开展社会主义荣辱观教育，开展“感动闽北十大人物”评选活动，评选表彰一批闽北道德模范，群众性精神文明创建活动继续深化。全面开展“五五”普法工作，深化“平安南平”创建活动，7个县（市）被评为省级平安县（市），102个乡镇（街道）和1705个村（居、社区）达到平安建设标准。群众对社会治安的满意率达96.16%%，信访总量下降6.71%%。深入开展重点行业和领域的安全生产专项整治，没有发生较大安全生产事故。产品质量和食品安全专项整治行动取得阶段性成果。实施闽江流域水环境综合整治工程，开展小流域综合治理试点工作。加强生态公益林保护和绿化工作，森林覆盖率74.7%%。南平城区生活污水处理率65%%，生活垃圾无害化处理率92%%，规模畜禽养殖场污染治理取得阶段性成效。年初确定的节能减排目标可以完成。国防动员、人民防空、预备役、民兵等工作有新进展，蝉联全国“双拥模范城”称号。

奋斗伴随艰辛，成绩来之不易。这是省委、省政府和市委正确领导的结果，是人大和政协监督、支持的结果，是全市人民辛勤劳动的结果，是历届政府不断积累的结果。我谨代表南平市人民政府，向全市人民，向全体人大代表、政协委员，向各民主党派、人民团体、工商联、无党派人士和离退休老同志，向中央、省直驻南平机构和驻军、武警、公安干警，向包括港澳台同胞、海外侨胞、国际友人在内的所有关心、支持闽北发展的人们，致以崇高的敬意和衷心的感谢！

回顾过去一年，我们也清醒地看到存在的困难和问题。一是发展不足，经济总量偏小。地区生产总值、规模工业等主要经济指标增幅跟不上全省步伐，大项目特别是生产性大项目不多，产业结构尚不合理，农业产业化水平有待提高，工业突出了但未突破，旅游优势没有充分发挥。二是基础薄弱，加快发展受到制约。高速公路建设滞后，铁路、机场设施亟待提升，部分区域电网网架较为薄弱，缺少电源支撑点；城市规划比较滞后，基础设施欠账较多，管理水平较低，经营运作不活。三是财力有限，统筹能力不强。农村基础脆弱，防灾减灾能力有待加强；社会事业发展滞后，欠账较多；社会保障体系仍不健全，部分群众生活还比较困难，就业、就医、就学、住房等领域需要解决的问题较多；生态建设、环境保护、节能减排压力增大；社会不稳定因素较为复杂，安全生产形势不容忽视。四是全民素质有待提高，人才外流较为严重，后备劳动力教育培训还存在差距。五是政府职能转变滞后，自身建设有待加强。干部队伍解放思想的力度还要进一步加大，抢抓机遇的意识还要进一步增强，闯拼发展的精神还要进一步强化，跃升发展的信心还要进一步树立。体制障碍尚需突破，运作能力还要增强，工作责任心、行政执行力亟待提高，政府工作还存在越位、缺位、不到位的现象。我们要高度重视这些问题，并认真加以解决。

　　二、海西绿色腹地跃升发展的目标任务

党的十七大提出全面建设小康社会新要求，省委八届三次全会作出海峡西岸经济区建设新部署，市委三届四次全会描绘了海西绿色腹地的发展蓝图，科学调整我市全面建设小康社会的

目标。这是基于“海峡西岸”上升为中央决定和国家战略，闽北必须融入大局，勇当前锋；这是基于全省加快建设科学发展先行区和两岸人民交流合作先行区的要求，闽北必须抢抓机遇，先行先试；这是基于我省各地及周边地区竞相发展的形势，闽北必须加速崛起，做大总量；这是基于我们经过积累积淀，“十一五”主要预期目标可望提前完成的现实，闽北必须更大作为，跃升发展。

根据党的十七大精神和市委的部署，我市“十一五”规划和本届政府任期目标调整为：到2010年，即“十一五”末，地区生产总值突破700亿元，增加150亿元，年均增长13.8%，提高4.3个百分点；财政总收入达61.5亿元，增加21.5亿元，年均增长19.1%，提高9.8个百分点；城镇居民人均可支配收入18100元，增加3600元，年均增长13%，提高5个百分点；农民人均纯收入达6300元，增加720元，年均增长9%，提高2.5个百分点。到2011年，即本届政府任期届满，地区生产总值达800亿元，年均增长13.8%；财政总收入达70.6亿元，年均增长17.7%；城镇居民人均可支配收入20500元，年均增长13%；农民人均纯收入6860元，年均增长9%。到2012年人均生产总值超过30000元，比2000年翻两番，到2017年与全省同步实现全面建设小康社会目标。实现这一目标，主要任务是：

构建海峡西岸绿色腹地。保护好闽北青山绿水，发展绿色经济，促进资源永续利用，把生态优势转化为竞争优势，实现闽北加快崛起，成为海西生态腹地。科学开发利用优势资源，培育发展旅游、生物、养生、创意等特色产业，提升传统产业，加速产业集聚，做大经济总量，成为海西经济腹地。依托广阔的发展空间，做大做强、做精做美南平中心城市，着手谋划新兴中心城市和新兴工业基地，加快县域中心城市建设，发展闽北城镇群，成为海西后发腹地。

构建南接北联战略通道。着眼于加强中心城市组团间的联系，建设80公里沿江快速通道，改造和新建14座桥梁，并与城市道路、高速公路网有机结合，形成中心城市交通网。着眼于推进闽北与周边大中城市的对接，全面建设列入国家高速公路网和海西高速公路网的9条高速公路，争取本届政府任期内70%%建成通车，形成闽北高速公路网。着眼于参与区域经济分工与协作，改造提升既有铁路，争取京台、沪杭广、景宁等快速铁路穿过南平，汇集建阳、武夷山之间，与高速公路枢纽相呼应，同时扩容升级武夷山机场，形成闽北与全国各大区域的立体交通网。

构建连片发展前锋平台。做大闽北产业集中区、南平工业园区、荣华山产业组团，大力推进各具特色的县市产业园区建设，提高空间集中度、产业集中度和行业关联度，加速企业向园区集中，尽快形成规模，打造产业集聚的基地。突出北联优势，利用周边地区产业发展的空间，优化投资环境，促进人流、物流、资金流、信息流的形成，集聚更多有利要素为闽北所用，成为发达地区产业转移的洼地，打造对外开放的载体。科学设置入园门槛，明确园区功能分工，注重选资选业，锁定重点产业，走新型工业化道路，促进经济发展方式转变。着力推动与台湾农业、旅游业和高新产业的深度对接，提升对台合作水平，打造先行先试的窗口。

实现跃升发展，是一个创新创业创造、务实落实真实的过程。必须保持“好”的态势。全面融入海西大局，转变发展观念，增强发展信心，敢拼敢闯，体现作为。正确把握闽北发展的阶段性，坚持好字优先、快字为要、稳字为重，寓好于快中，寓快于实中，寓实于重中，注重投资拉动下的消费、出口拉动相协调，注重二产带动下的三次产业协同发展，实现速度、质量与效益同步提升，保持持续发展的形势、趋势、态势和气势，力争全市经济持续又好又快发展。

必须发挥“锋”的作用。坚持全市“一盘棋”，10个县（市、区）形成整体，共同肩负起纵深推进的重大使命。强化沿海意识，发挥日益凸显的海西效应，利用日益改善的发展条件，加强与长三角和中西部地区的经济协作。中心城市、邵武市和浦南沿线县市必须加速崛起，成为支撑闽北经济总量和追赶先进地区的主力军，顺昌、光泽、松溪、政和要打牢基础，加快蓄积发展力量。

必须做足“通”的文章。坚持能快则快，做细做深交通项目前期工作，盘活城市资源、资产和资金，筹措建设资本金，举全市之力打开大通道。积极创造条件，建设火电、核电等新的能源点，加快建设输变电设施，完善电网网架。培育发展金融产业，扩大金融战略性合作，精心策划融资项目，拓宽融资渠道。着眼于降低企业发展成本，把“通”字放在园区建设首位，抓好基础设施配套，建设标准厂房，创造企业入园“直通”条件。建立政府、企业良性互动关系，开辟便民便商“绿色通道”。

必须突出“民”的利益。实施扩大就业发展战略，大力发展教育卫生事业，推动文化繁荣发展，致力发展民生。将更多新增财力向民生倾斜，提高民生支出占财政支出的比重，致力改善民生。围绕“学有所教、劳有所得、病有所医、老有所养、住有所居”，适时出台惠民政策，积极发展社会福利和慈善事业，致力保障民生。

必须营造“和”的氛围。调动各方面的积极因素，激发全社会的创造力。从解决发展中的突出矛盾和问题入手，积极推进体制创新，增创发展新优势。完善公共财政体制，提高社会管理和公共服务的水平。注重可持续发展，加强资源节约和环境保护，实现人口资源环境相协调。关注弱势群体，维护公平正义，保持社会安定稳定，促进社会和谐发展。

三、扎实做好2008年工作

今年政府工作的指导思想是：全面贯彻党的十七大精神，高举中国特色社会主义伟大旗帜，以邓小平理论和“三个代表”重要思想为指导，深入贯彻落实科学发展观，按照省委“四谋发展”的实

践主题、“四个重在”的实践要领、“四个关键”的工作要求，落实市委三届四次会议的工作部署，实施项目带动、品牌带动，坚持着力民生、着力民心，加快建设科学发展的先行区、两岸人民交流合作的先行区，进一步发挥纵深推进的前锋作用，加快实现闽北跃升发展。

2008年，全市经济社会发展的主要预期目标是：地区生产总值550亿元，增长14%；规模工业515亿元，增长25%%；财政总收入46.64亿元，增长14.8%，地方一般预算收入27.52亿元，增长15.2%；城镇居民人均可支配收入14870元，增长13%，农民人均纯收入5470元，增长9%；全社会固定资产投资增长20%；外贸出口增长12%，实际利用外资增长10%；城镇登记失业率控制在4.5%以内；社会消费品零售总额增长14%；人口自然增长率控制在7‰以内；单位生产总值能耗降低4.3%，二氧化硫排放量下降1.97%，化学需氧量减少0.67%。在实际执行中力争更好更快更实。

（一）推进工业实质性突破

做大产业园区。闽北产业集中区重点推进童游机械电子组团和林产工贸园区建设，南平工业园区抓好大横绿色产业区和夏道组团基础设施建设，荣华山产业组团着力抓好启动区开发。每个组团分别完成征地5000亩，建成标准厂房30万平方米以上，基础设施投资3亿元，入园企业实际投资5亿元，招商引资8亿元。抓紧启动建设武夷山软件园区和创意产业园区，鼓励和引导新兴产业发展。

培育新“五南”式企业。对已列入培育对象的31家重点工业企业实行滚动管理，帮助企业制定扩张发展的目标措施，引导有条件的企业上市融资，进行资本运作，低成本扩张。扶持有条件的企业设立国家级、省级研发中心，鼓励参与国家或国际、国内行业标准制定。引导企业建设企业文化，引入现代经营理念，提升企业发展竞争力。坚持抓大扶小，培育有潜力的小企业增资扩营滚动发展，帮助企业解决实际困难，使之成为后备力量。

发展优势产业。在提升七大产业的同时，着力培育旅游、生物、养生、创意等特色产业，上半年完成产业发展规划，出台配套政策，确定发展方向和重点。生物产业、创意产业、养生产业、软件业重在引进和开发重大项目；竹木加工立足于整合提升，扶优限劣；全面开展矿产资源普查，做好矿产业发展规划，并与大公司联合进行矿产资源综合深度开发。根据产业发展的基础和优势，策划项目、生成项目和落实项目。对列入重点开发的项目，实行项目责任制，有序推进，尽快形成新兴产业体系。

（二）增强农业和农村经济发展活力

推进现代农业发展。围绕农业发展、农民增收，加强农业基础建设，转变农业发展方式。突出一县一特色、一乡一业、一村一品，做大木竹、茶果、畜牧三大主导产业，加大力度发展烟业，引导扶持发展区域特色经济。稳定粮食播种面积，确保粮食生产稳定。加强农业技术推广体系建设，充分发挥科技特派员作用，积极探索农业科技成果进村入户的有效机制和办法。推广无公害、绿色、有机农产品生产，保证农产品质量安全。培育一批优势品牌，依法保护注册商标、地理标志和知名品牌。集中扶持发展一批骨干龙头企业，争取新增规模以上农业产业化企业50家。依照《农民专业合作社法》，出台配套政策，鼓励和引导农民建立专业合作经济组织，力争达到1000家。

做好新农村建设规划。坚持规划先行，以县（市、区）为单位组织专业队伍，制定农村经济发展、村镇建设、劳动就业、基层组织建设四个规划。经济发展规划重在因地制宜，宜农则农、宜工则工、宜商则商，统筹产业布局。村镇建设规划重在合理配置土地资源，统筹生产、居住、社会事业和农村基础设施建设。劳动就业规划重在农村劳动力培训，提高素质，促进就业，增加务工收入。基层组织建设规划重在增强农村干部队伍活力，提高民主管理水平。对区位优越、资源基础好的中心村和中心集镇要优先予以规划扶持。

加强农村基础设施建设。逐步提高农村公共服务水平，进一步改善农村生产生活条件。实施农业综合开发土地整理、改造中低产田、建设商品粮基地和标准农田11万亩，完成烟叶基础设施建设1.72亿元；建设林业“四大基地”36万亩，新开竹山机耕路1000公里，新建防火林带1300公里，加快竹山灌溉项目建设。新增农村公路硬化400公里，加强农村道路安全管理，发展农村公共交通，新增通客车行政村50个。实施小流域综合治理和源头防洪体系建设，组织实施库区项目。加快大型灌区节水改造，推广农业节水设施和技术。除险加固水库20座，建成山地水利4万方、村级供水150处。新建户用沼气池8700口。新建和改造电网项目11个。建设15个乡镇、200个村垃圾处理场。

不断深化农村改革。进一步完善南平机制，强化互动联动，规范“八大员”队伍管理。完善农村基本经营制度，切实稳定农村土地承包关系，依法落实和维护农民承包土地的各项权利。坚持依法自愿有偿的原则，鼓励土地承包经营权流转。严格土地征收征用、农村集体建设用地流转、宅基地整理等的审批管理，切实保护农民土地权益。深化集体林权制度改革，妥善解决遗留问题，大力推行生态公益林管护和林业管理服务新机制，总结推广林权抵押贷款、森林保险、股份合作经营等经验。继续推进乡镇机构和县乡财政体制改革，完善农村税费改革等配套改革。加强农村信用体系建设，开展村镇银行试点，改善农村金融环境。

（三）提升以旅游业为龙头的第三产业发展水平

全力打造旅游经济龙头。按照建设武夷山国际性旅游度假城市的要求，推进机场扩容升级、国际会展中心、山水实景演出、A级体育赛事场地、自驾车旅游服务基地、自然博物馆、历史博物馆和养生基地等项目建设。实施主景区畅通工程，完善、开辟景区旅游线路。加强自然保护区保护与管理，扩大保护范围。高标准做好度假区规划，重点布局大型旅游项目。加强全市旅游资源管理与保护，有效控制和科学开发战略性旅游资源，从整体上制定规划、整合资源、打造品牌、对接线路，加快形成大武夷旅游经济体系。加快建设茫荡山、延平湖、和平古镇、天成奇峡、仙沐园温泉等重点旅游景区。开辟红色旅游线路。强化与宁德、三明、衢州、金华、上饶、黄山等区域旅游协作，深化武夷山与厦门、杭州旅游合作，提升武夷山与阿里山旅游协作层次。进一步拓展旅游市场，做好旅游营销，提升旅游品牌，丰富旅游产品，保持旅游收入高于旅游人数增长。

加快物流业和服务业发展。制定现代物流业发展规划，力争启动西芹物流园区建设，编制邵武、闽北产业集中区物流园区规划。推行电子商务、连锁经营和物流配送等新型流通方式，建立专业化、社会化物流服务网络。整合提升商贸、运输、旅游、餐饮等传统服务业，鼓励发展市场推广、产品检测等生产性服务业，培育壮大汽车、体育健身、卫生保健、社区服务等新消费增长点，加快发展中介信息和法律服务等新兴服务业。培育一批重点农产品专业市场，建成20个新型乡镇商业区、20个社区商业服务中心和200个农村信息服务站。

培育壮大金融产业。把金融业作为一个重点产业加以培育，发挥现有商业银行作用，引进股份制银行设立分支机构，筹建地方性金融机构，做大做强农村信用社。加快银行、保险、证券业发展，加强诚信体系建设，优化金融服务，做好金融监管。规范培育典当、担保业。加强政策性金融合作，不断满足经济发展多元化的金融需求。

（四）实施项目带动和品牌带动

强化重点项目建设。初步安排市重点项目123个，其中在建81个，预备31个，前期11个，总投资764亿元（不含前期重点项目），当年计划投资105.8亿元，增长20%%。积极策划生成一批基础性、生产性大项目，争取更多项目列入省重点。加快浦南、武邵高速公路建设，确保浦南高速公路年底通车，力争宁武、松建、龙浦高速公路年内动工，做好建古、南平绕城、邵光及延平、来舟至顺昌高速公路前期工作。开工建设向莆铁路南平连接线、南平东支线改线、峰福线横南段铁路电气化技改。抓好南平城区铁路改造和京台、杭广、景宁、建武铁路南平段规划等项目前期工作。加快建设南平500千伏输变电工程，抓紧建设南平九越二期、大横、邵武安平二期、建瓯东安、浦城丹桂220千伏及南平安丰、江南、西区尤坑、邵武大埠岗、建瓯徐墩、建阳水吉、浦城九秋、九牧、顺昌新屯等110千伏输变电工程，按照属地管理做好项目服务，确保电网建设项目落地。推进邵武火电厂技改，筹建闽北火电厂。争取完成核电项目初步可行性研究论证，开展工程可行性研究报告编制工作。

增强招商选资实效。抓好招商平台建设，办好“5·13”投洽会、旅游文化节、海峡两岸茶博会等重大活动，积极参加“6·18”、“9·8”等重大展洽会，深化泉南山海协作以及闽东北五地市、四省九市、四省十四市的区域合作，做好项目对接，提高引进项目质量和效益。充分发挥侨联、台联、台属联、旅港、旅澳联谊会、各地商会等组织的作用，加强与国外、港澳台地区经济技术合作。调整优化出口商品结构，扩大对外贸易和劳务输出。

注重品牌带动。强化争先创优意识，把品牌打造作为区域经济发展的整体行动，扶持企业打造品牌，提高品牌的科技和创意含量。以品牌带动提升产业发展，重点培养产品品牌、企业品牌、行业品牌、城市品牌，丰富品牌内涵。力争全市新增中国驰名商标、中国名牌产品、国家免检产品各2个，地理标志证明商标2个，新增福建著名商标和福建名牌产品各12个。

　　（五）建设富有闽北特色的山水城市

加快南平中心城市建设。按照建设山水园林城市的要求，统筹南平中心城市各组团要素的衔接，合理布局城市功能，完成总体规划修编。充实项目

班子，落实项目责任，确保首批26个城建重点项目按计划实施。加快城区连接线、沿江快速通道一、二期工程、八仙大桥、剑州大桥、西城大桥、沿江景观带、成功路等项目建设。积极推进九峰山、玉屏山公园改造及“三泰二建铁中”和东坑片区改造。继续策划实施第二、三批城建项目。树立城市经营理念，推进公交、环卫、园林绿化等市政公共服务市场化运作。理顺市区管理体制，建立长效管理机制。

谋划新兴中心城市。按照市委关于打造新兴中心城市和新兴工业基地战略部署，组建专门工作班子，迅速启动前期筹备工作，开展调研论证。结合闽北产业集中区开发、武夷山旅游产业发展和交通网络建设，统筹新兴中心城市的基础设施、产业发展和城市规划总体布局，为未来发展打下基础、留足空间。

发展县域中心城市。支持做大县域中心城市，长远规划，分步实施，有序推进，协调配套，彰显个性。武夷山按国际性旅游度假城市高标准建设，邵武按二级中心城市提升品位，建阳、建瓯、浦城重在城市规模扩张和集聚，顺昌、光泽、松溪、政和实施“小县大城关”战略，推进城镇化进程。

（六）统筹发展社会事业

优先发展教育。加快实现“双高普九”和普及高中阶段教育，重点发展职业技术教育，逐步提升高等教育，全面推进素质教育，重视学前教育和特殊教育。制定出台教育优先发展规划及其配套政策，提高农村义务教育阶段中小学生均公用经费定额标准，继续做好外来务工人员子女就近就学工作。继续建设农村中小学现代远程教育工程，落实城乡学校与教师对口帮扶。整合教育资源，调整学校布局。建设南平一中江南分校，积极争取利用原林学院校区办学，加强薄弱校建设。加强职教重点专业和实验实训基地建设，支持武夷山职业学院和闽北卫校建设，加快福建林业职业技术学院、闽北职业技术学院新校区建设，确保如期建成投入使用。支持武夷学院提高办学水平。

推进科技进步。强化企业技术创新主体地位，积极引导企业加大研发投入。整合科技资源，加强企业与高等院校和科研机构协作，加强科技成果对接，逐步形成以科技进步和创新为基础的竞争优势。推广新技术成果200项，开发新产品200项。建设省级电线电缆产品质量监督检验中心，力争年底投入使用。进一步加强科普工作，提高公民的科学素质。培养和引进一批掌握核心技术的人才、复合型科技人才和高技能人才。

促进文化繁荣。大力宣传闽北历史文化，重点弘扬朱子文化，重视发掘保护文化遗产，发展特色文化。推进文化体制改革。加大公益性文化事业投入，重点向乡村和城市社区倾斜，扩大公共文化服务覆盖面，建成南平大剧院、南武体育馆和博物馆。进一步繁荣新闻出版、文学艺术和社会科学，加强网络、娱乐场所、音像书刊市场监管整治。启动有线数字电视整体转换，实现全市80%%的村广播电视光缆联网，完成高山台无线覆盖工程。以2008年奥运会为契机，广泛开展全民健身运动，推进群众体育和竞技体育协调发展，提高人民群众身体素质。

加强卫生和人口与计生工作。重视改善农村医疗卫生条件，加快农村卫生技术人才培养，提高农村卫生技术人员待遇。合理布局城区医疗网点，大力发展社区卫生服务，南平城区、邵武、武夷山、建瓯、建阳和有条件的县社区卫生服务中心覆盖率达100%%。深化医疗机构管理机制改革，开展医院评价和等级医院评审。提高重大疾病防控和突发公共卫生事件应急处置能力，抓好计划免疫、职业卫生、妇幼卫生保健工作。加强人口和计划生育工作，推进生育关怀行动，稳定低生育水平，提高出生人口素质，综合治理出生人口性别比升高等问题。

（七）提高人民生活水平

扩大就业和再就业。健全城乡就业服务体系，落实就业再就业政策，帮助城镇“零就业家庭”和就业困难人员实现就业，创建充分就业社区。城镇新增就业2.4万人。健全农民工就业服务制度，农村劳动力转移就业5万人。全面实行劳动合同和集体合同制度，积极做好高校毕业生、退伍转业军人、残疾人就业工作。做好就业培训、维权和信息服务，构建劳动保障服务平台。

完善社会保障。扩大企业职工养老、失业、工伤和生育保险的覆盖面，城镇职工基本医疗保险、城镇居民基本医疗保险和新型农村合作医疗参保人数分别达到34.3万人、35万人和205万人。落实城乡居民最低生活保障制度，适当提高标准。探索做好被征地农民的社会保障工作。加大对革命老区、贫困地区、民族地区、边远地区和库区的扶持力度。加强老龄工作，保护妇女儿童合法权益，发展以扶老、助残、救孤、济困为重点的社会福利和慈善事业，提高社会救助水平。

办好为民惠民实事。城乡教育方面，免除城市和农村义务教育阶段学生学杂费，免费为农村义务教育阶段学生提供教科书，补助农村义务教育阶段寄宿生生活费，新建农村寄宿制学校宿舍20座，改造中小学危房50项，实施高等院校、中等职业学校学生补助政策和困难学生资助政策。医疗卫生方面，改造提升15个乡镇卫生院，提高新型农村合作医疗补助标准，进一步开展“光明行动”，继续实施农村卫生“123”工程，大力提高免费婚检率。文化建设方面，改造完善乡镇综合文化站，深入开展农村“家园清洁行动”，推进“通信信息化助推新农村”项目建设，完成20户以上700个自然村迎奥运广播电视村村通，建设社区邮政便民服务站500个、海西书报亭80个。社会保障方面，深入实施农村劳动力转移培训就业工程，启动建设南平社会福利中心，新建和改建10所农村敬老院；建立以大病统筹为主的城镇居民基本医疗保险制度，建立独生子女伤残死亡家庭扶助制度。住房保障方面，逐步解决城市低收入家庭住房困难，对申请廉租住房租赁补贴的城市低保家庭基本做到应保尽保，规范经济适用住房制度，实施“造福工程”搬迁8000人，基本完成“侨居造福工程”。食品安全方面，继续治理“餐桌污染”，建设“食品放心工程”，巩固产品质量和食品安全专项整治成果。

（八）推进和谐社会建设

建设生态文明。严格执行国家土地政策，加强建设项目用地和批后管理，推进耕地开发和土地整理，确保耕地占补平衡。严把项目开发环保关，保护水源、土地、森林等自然资源。继续实施国家级生态示范区建设，完善生态公益林管护和补偿制度，加强公路、铁路、河流两侧一重山绿色长廊建设和保护，严格控制沿江沿河建设永久性建筑。科学开发利用资源，确保可持续发展。

加强节能减排工作。认真落实节能减排工作责任制，坚决淘汰落后生产技术，坚决遏制高耗能、高排放行业发展，推进重点行业、重点耗能企业节能技术改造，鼓励发展节能产业和循环经济，降低单位生产总值能耗。加强环境监管和执法力度，控制主要污染物排放总量。继续实施闽江流域水环境综合治理，抓好各类污染源治理，加大规模畜禽养殖场污染治理，巩固禁建区治污成果，全面开展适度养殖区治理。推行工业园区污染集中处理。加快垃圾焚烧发电和城市污水处理产业化项目建设，力争今年开工建设邵武、建阳、浦城三处城市联盟及南平中心城市垃圾焚烧发电厂，建成邵武、建阳、建瓯、顺昌污水处理厂，开工建设浦城污水处理厂，完成光泽、松溪、政和污水处理厂前期工作。

加强精神文明和民主法制建设。不断加强社会主义荣辱观教育，努力构建社会主义核心价值体系。深化公民道德实践活动，以“迎奥运、讲文明、树新风”为主题，广泛开展群众性精神文明创建工作，着力建设诚信政府、诚信社会。深入开展“五五”普法，提高全民法制意识，拓展法律服务，推进依法治市进程。重视青少年思想道德教育，实施关爱工程，积极构建社会、学校、家庭三位一体的预防犯罪体系，进一步完善预防青少年违法犯罪工作组织体系和工作机制。加强民族宗教工作。扩大基层民主，完善民主管理制度。发挥城乡基层自治组织和社团、行业组织作用，做好城市社区工作。

建设“平安南平”。加强社会治安综合治理，抓好平安创建系列活动。坚持打防并举，严厉打击各种犯罪活动，不断健全治安防控体系，提高人民群众安全感。完善社会管理与服务，做好信访工作，重视人民调解，健全矛盾纠纷排查调处机制，积极预防、妥善处置影响社会稳定的事件。严格落实安全生产责任制，预防和遏制重特大安全生产事故发生。建设应急处置体系，健全事故灾难和公共安全预警机制，有效应对自然灾害和突发事件。

支持国防和军队建设。强化国防教育，增强全民国防意识，完善国防动员机制，扎实做好国防动员工作，支持部队战备训练和后勤保障。加强预备役部队、民兵建设，全面推进人防体系建设，落实优抚政策，认真做好城镇退役士兵、军转干部和随军家属安置工作，巩固军政团结，提高双拥共建水平。

　　四、努力提高领导科学发展的能力

面对建设海西绿色腹地的繁重任务，必须切实加强政府自身建设，努力提高领导科学发展的能力。

　　强化学习，提高素质。深入学习十七大精神，深刻领会科学发展观内涵，掌握科学发展的根本方法，把握科学发展的规律，提高推进跃升发展的能力。努力学习现代经济知识、金融知识，运用金融工具，拓宽融资渠道，研究影响发展方式转变和经济结构调整的主要因素，提高驾驭市场经济的能力。努力学习科学技术知识和社会管理知识，善于在推进经济发展的同时兼顾各方的发展要求，提高统筹协调的能力。加强统计基础工作，开展第二次全国经济普查，提高宏观经济形势预测预警和应对能力。

更新观念，注重运作。大力倡导闯拼精神，不等、不靠、不让，不放失每一个机遇、不忽视每一分积累。正确理解中央有保有压、区别对待的宏观经济政策，把握扶持农业、倾斜后发展地区、支持重大基础设施建设的导向，抢抓机遇，主动作为，加快追赶步伐。用好、用足、用活国家部委和中央企业支持海西发展的各项政策，做好项目前期准备工作，争取上级在项目、资金、指标上的支持。按照国家产业政策和外资投资指导目录，做好项目对接，提高项目运作能力和实际效果。

创新体制，转变职能。正确处理政府与市场、社会和公民的关系，深化行政体制改革，推进行政结构重组、流程再造、手段更新、职能转变。认真解决政府职能存在越位、缺位、不到位的问题。成立行政服务中心，深化审批制度改革，整合部门审批职能，进一步清理、减少妨碍市场开放和公平竞争的审批事项和环节，规范办事程序，提高行政效率，降低行政成本。探索行政综合执法改革，提高政府行政监督效率。加快事业单位改革，培育社会中介组织，推进城市公用事业改革，更好地履行政府公共服务和社会管理职能。完善政府绩效评估，深化创业竞赛，强化各项工作定性定量的约束性要求，提高政府执行力。

依法行政，维护公平。严格依照法定权限和程序行使政府职权，进一步落实依法行政实施纲要，加强和改进行政执法，健全执法责任制，切实维护公民、法人和其他组织合法权益，提高政府公信力。主动接受人大及其常委会的法律和工作监督、政协和各民主党派的民主监督，以及各界群众和新闻媒体的社会监督。进一步健全科学决策、民主决策制度，高度重视人民群众、专家学者的意见，出台涉及民生的重大政策，实行听证制度，征求各方意见，依法保障群众的知情权、参与权、表达权和监督权。完善政务公开制度，重点推行公用事业单位办事公开。贯彻实施政府信息公开条例，及时公开人民群众关心、关注的信息，畅通政府与群众双向沟通渠道，促进政府工作公正、公平、便民。

从严治政，清正廉洁。认真执行中央、省委和市委关于廉洁从政的规定和要求，坚持教育、制度、监督并重，从权、钱、物等关键环节入手，从源头上防治腐败，深化政府系统反腐倡廉工作。各级政府和各部门领导要切实履行“一岗双责”，认真落实主要负责人述职述廉制度。强化审计监督和行政监察，加大对重点领域、重点部位和关键环节的监督。特别是今年开工建设项目多，必须关口前移，有效防范腐败现象发生。党政机关车辆要带头缴交过路费，严格执行行政机关统一津贴补贴有关规定，严禁乱发奖金、津贴、补贴、过节费或变相发放钱物。牢记“两个务必”，依法履职，秉公用权，端正政风，树好形象。

各位代表，让我们高举建设中国特色社会主义伟大旗帜，在市委的领导下，团结和依靠全市人民，坚定信心，锐意进取，奋力拼搏，为加快推进海西绿色腹地跃升发展而努力奋斗！
